# Supplementary material for: Aqueous Extracts from Hemp Seeds as a New Weapon against Staphylococcus epidermidis Biofilms
Source: Int J Mol Sci. 2023 Nov 7;24(22):16026. doi: 10.3390/ijms242216026 (PMC10671263; doi:10.3390/ijms242216026)
Supplement: Supplementary file 1 [file ijms-24-16026-s001.zip › ijms-2677226-supplementary.pdf]

Supplementary Data

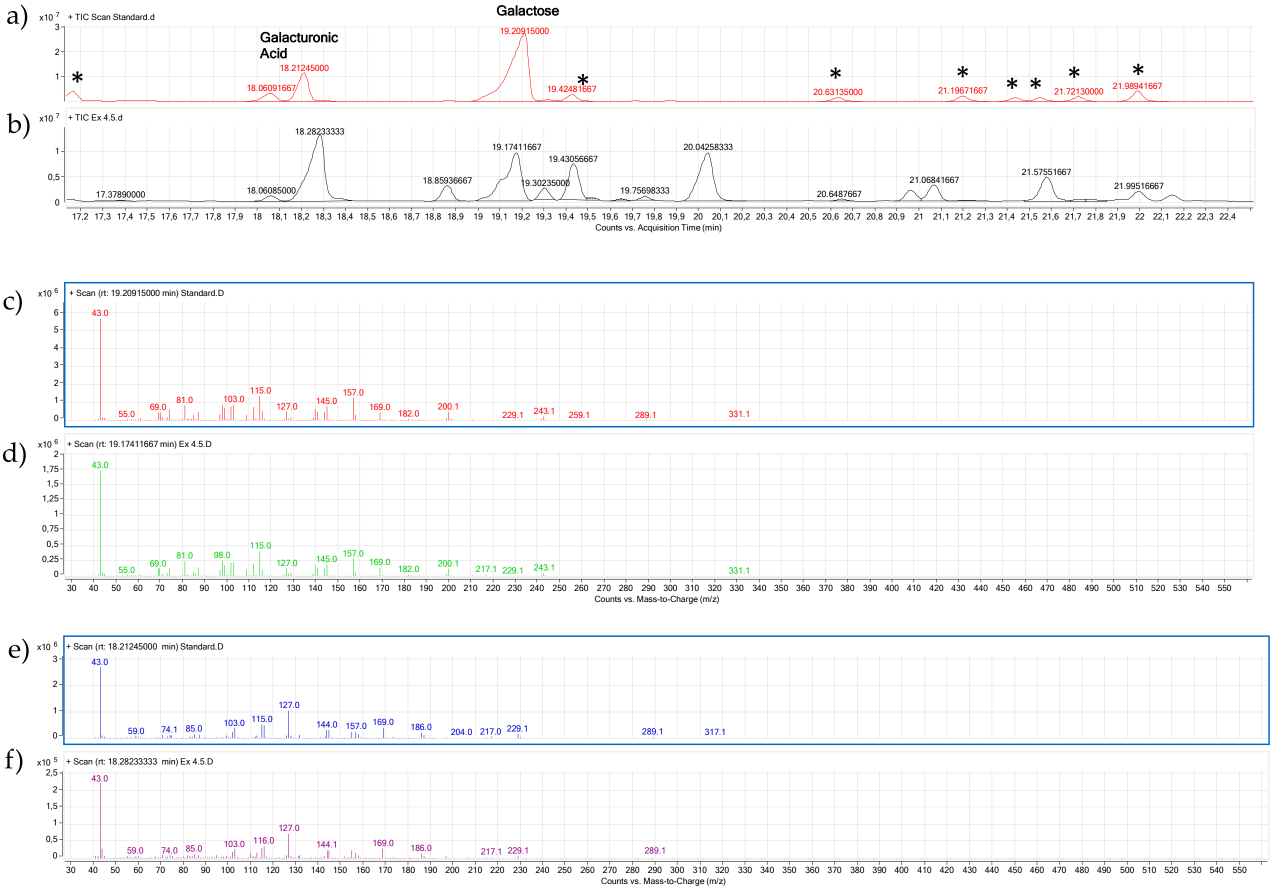

Figure S1. a) Selected region of the TIC (total ion chromatogram) of the standard containing the Galactose and the Galacturonic acid. b) the same region for the Ex 4.5 extract; c) and d) mass spectra of the Galactose standard and the Ex 4.5, respectively. e) and f) mass spectra of the Galacturonic standard and the Ex 4.5, respectively. The region of the chromatograms has been chosen as an example. In the same way, all the components have been recognized for all the extracts. Peaks marked with (*) referred to impurities of the reaction.

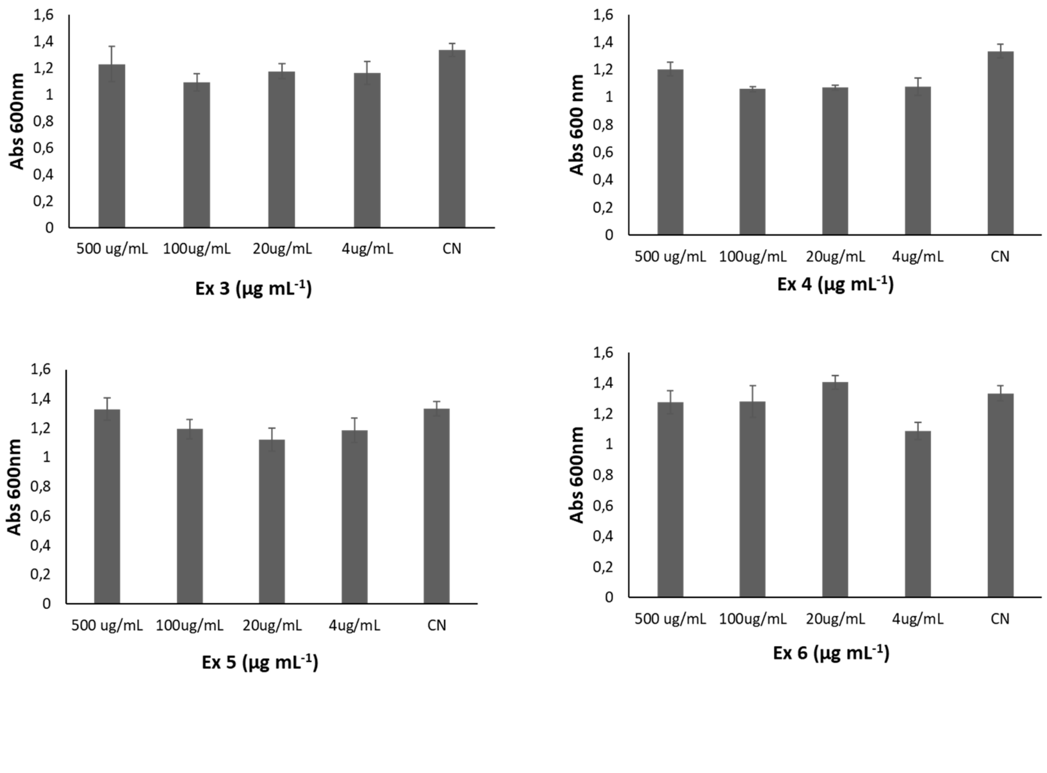

Figure S2. Minimal Inhibitory Concentrations (MIC) of the extracts at different concentrations against *S. epidermidis* RP62A compared to untreated bacteria (CN).

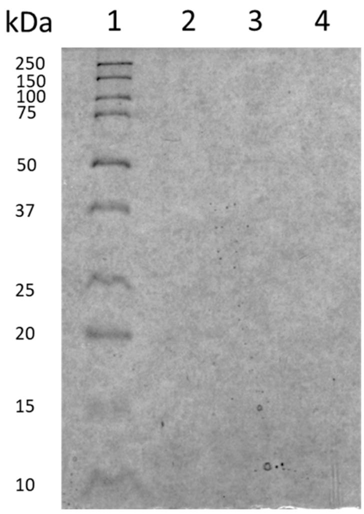

Figure S3. SDS-PAGE analyses stained by Blue Coomassie. Lane 1: molecular weight markers; lane 2: Ex7d; lane 3 Ex7; lane 4: Ex4.5.

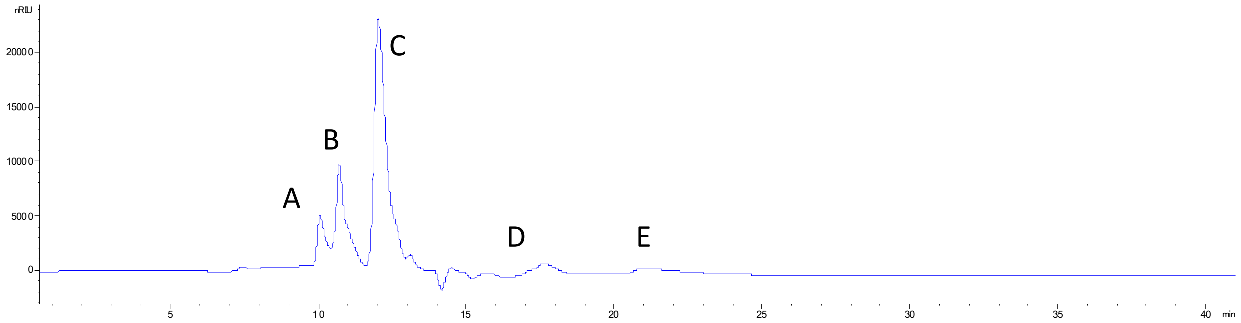

Figure S4. HPLC chromatogram of Ex7d.
